# Demandingness and Public Health Ethics

**DOI:** 10.1515/mopp-2018-0057

**Published:** 2019-05-01

**Authors:** Alberto Giubilini, Julian Savulescu

**Keywords:** demandingness, public health ethics, duty of easy rescue, vaccination, antibiotic resistance

## Abstract

Public health policies often require individuals to make personal sacrifices for the sake of protecting other individuals or the community at large. Such requirements can be more or less demanding for individuals. This paper examines the implications of demandingness for public health ethics and policy. It focuses on three possible public health policies that pose requirements that are differently demanding: vaccination policies, policy to contain antimicrobial resistance, and quarantine and isolation policies. Assuming the validity of the ‘demandingness objection’ in ethics, we argue that states should try to pose requirements that individuals would have an independent moral obligation to fulfil, and therefore that are not too demanding. In such cases, coercive measures are ethically justified, especially if the interventions also entail some benefits to the individuals; this is, for example, the case of vaccination policies. When public health policies need to require individuals to do something that is too demanding to constitute an independent moral obligation, states have an obligation to either provide incentives to give individuals non-moral reasons to fulfil a certain requirement — as in the case of policies that limit antibiotic prescriptions — or to compensate individuals for being forced to do something that is too demanding to constitute an independent moral obligation — as in the case of quarantine and isolation policies.

## 1 Introduction

Public health policies often require individuals to make personal sacrifices for the sake of protecting other individuals or the community at large. These sacrifices can vary from minimally demanding to very demanding ones. In the context of public health, we can conceive the demandingness of a policy in terms of the individual costs it involves with regard to the harm or risk of harm it imposes on individuals; admittedly, it can sometimes be difficult to determine the level of demandingness of certain requirements, and we would often need to rely on intuitions (we will return to this point below). If we accept the ‘demandingness objection’ in ethics, then arguably individuals do not have any moral obligation to contribute to public health through excessively demanding personal sacrifices, unless they are legitimately coerced by the state — that is, they do not have an *independent* moral obligation to make very demanding personal sacrifices. The ‘demandingness objection’ is, roughly, the idea that if a requirement is too demanding, or supererogatory, it can’t constitute a *moral* obligation^1^ (see e. g. Braddock 2013; Hooker 2009). However, when coercive and very demanding public health policies are legitimate and necessary (for example, to prevent epidemics of infectious diseases), individuals arguably do have the independent prima facie ethical obligation to obey the law. In this paper, we argue that in order for public health policies to be ethically justified, they should either coerce people into doing what people would have an independent moral obligation to do anyway, and therefore what is not too demanding and can contribute to important public health goals, or provide incentives and/or compensation when their demandingness is such that individuals would not have an independent moral obligation to fulfil the requirement. This is the case of supererogatory requirements, at least according to the standard ‘Sacrifice View’ of supererogation whereby a good action is supererogatory if it is too costly for an agent to be morally required (see e. g. Ackeren 2018). While alternative understandings of supererogation exist (for an overview, see Archer 2018), the Sacrifice View is the most adequate for the purpose of our paper as we are interested in assessing what levels of sacrifice the state can permissibly impose on an individual with and without incentives and compensation. Because it can be difficult to establish the level of demandingness of certain requirements, it can be difficult to determine whether a certain requirement is supererogatory either. Here, we will focus on the three examples of public health policies that seem to entail different levels of demandingness.

The first is an example at the non-demanding end — what we can call the ‘easy rescue’ end. Certain countries have implemented different types of coercive vaccination policies that require people to vaccinate their children against certain infectious diseases; if vaccination counts as a sacrifice at all (which is very questionable), it normally is a very small one: vaccination does entail a very small cost in terms of inconvenience and a very small risk of side effects, but it is very often beneficial to the vaccinated individual and, most importantly from the point of view of public health, prevents an individual from infecting others and contributes to the important public good of herd immunity. There is herd immunity when a sufficiently high proportion of the population is vaccinated against a certain infectious disease, so that the probabilities of contagion are so low that all the others are indirectly protected from that infectious disease. Herd immunity is important, for instance, to those who cannot be vaccinated because of some medical contraindications to certain vaccines (e. g. they are immunosuppressed), those who are too young to be safely vaccinated, and those for whom vaccination turns out to be ineffective. Demandingness in this case does not seem to represent a reason against the claim that there is a prima facie *moral* obligation to make one’s contribution to herd immunity by being vaccinated or by vaccinating one’s children. Vaccination seems to be a clear case of a moral duty of both collective and individual easy rescue (Giubilini et al. 2018).

However, other public health policies can be more demanding for individuals, and therefore make it more difficult to claim that individuals have an uncontroversial moral obligation to make personal sacrifices for the sake of other people’s good — aside from the independent moral obligation to fulfil any legal requirement. For instance, and this is our second example, antibiotic resistance has become a public health emergency (O’Neill 2016). In order to contain antibiotic resistance, it is necessary, among other things, to reduce the collective consumption of antibiotics, at least in certain areas. Some have suggested that, at least in certain countries, doctors might have to refrain in certain cases from prescribing antibiotics for certain self-limiting infections or might have to prescribe suboptimal antibiotic treatment in order not to erode the common good of antibiotic effectiveness (Foster and Grundmann 2006). It is not clear yet whether such an extreme measure would be necessary, but it is a real possibility. Arguably, enduring the inconveniences and the pain of a mild and treatable bacterial infection — such as urinary tract infection — is more demanding than getting vaccinated, to the point that it is questionable whether there is an independent moral obligation to forego antibiotics in certain cases. More importantly, there is a small but finite risk of developing overwhelming septicaemia and death from an inappropriately treated bacterial infection in such cases.

Our third example is quarantine and isolation measures. These are measures that require people who have been exposed to or infected by certain infectious diseases to be temporarily confined and to temporarily limit their social interactions and their liberty of movement, which seems to be a very demanding requirement. In the case of quarantine, there is the possibility that an uninfected individual contracts the disease from other infected, quarantined individuals or staff. It does not seem unreasonable to claim that quarantine and isolation are so demanding there is no independent *moral* obligation to be quarantined or isolated.

Many other examples of public health policies could be provided (lifestyle policies, such as those regulating drinking and smoking; travel policies; seat belt policies; hygiene policies; and so on). We will focus on the three examples provided, but our considerations can be extended to other public health policies as well, and towards the end of the paper we will say something about some other public health policies.

In all these cases, there are public and collective goods that seem to justify imposing more or less demanding requirements on individuals; and these public and collective goods are health related goods that arguably a state has the responsibility to guarantee. Therefore, there is a strong ethical case for implementing coercive policies, if such policies are necessary to guarantee such goods. Yet, if we assume that the demandingness objection is valid, individuals probably do have an independent moral obligation to be vaccinated, but it is harder to argue that individuals have an independent moral obligation to forego antibiotics in case of mild infections or to be quarantined or isolated. What are the policy implications of these different degrees of demandingness? Our claim is that the state is justified in implementing coercive vaccination policies, and perhaps ought to implement coercive vaccination policies, which means that they should punish those who fail to vaccinate. If restricting antibiotic consumption is necessary, the state should use incentives (financial or of other type) to give people non-moral reasons for not using antibiotics to treat certain infections in certain circumstances. Lastly, the state should compensate individuals who are quarantined or isolated.

We will proceed as follows. We will present some fundamental principles of ethics and public health ethics that have implications not only for whether certain requirements constitute independent moral obligations of individuals, but also for how public health policies should be designed in order to be responsive to demandingness. More specifically, we will show that there is a case for punishing failures to fulfil moral duties of easy rescue in public health: when a requirement also constitutes an independent moral obligation, there is a strong reason for coercing people into fulfilling that requirement through punitive measures. But there is also a case for incentivising overdemanding behaviours, when the good at stake is sufficiently important, and for compensating people who are forced to fulfil overdemanding requirements. Finally, in light of these more theoretical considerations, we will make some practical suggestions about how public health policies regulating vaccination, antibiotic consumption, and quarantine and isolation ought to be designed. We will also briefly mention other types of public health policies.

## 2 Ethics, public health ethics, and demandingness: Some general considerations

In this section we are going to provide some ‘conceptual tools’ that will be used in the remaining of the paper to support our arguments in favour of different types of public health policies.

### Demandingness and policies

Demandingness is an intrinsic aspect of morality. As put by de Lazari-Radek and Singer 2014, (p. 317), ‘morality asks us to do something that we may not want to do, or that may give rise to a conflict of motives’. However, there is a limit to the level of demandingness that morality can require, although different people have different ideas as to where exactly the threshold lies. To the extent that the state has a moral obligation to protect certain important public goods, it can be argued that it has a moral obligation to enforce policies that pose overdemanding requirements on individuals, if that is necessary to protect those important public goods. Herd immunity and a good level of public health are examples of important public goods. However, the power of the state to enforce coercive and demanding public health policies is constrained by what we take to be a basic ethical principle regulating policy making:

*The demandingness-policy principle*: the less demanding for individuals a coercive public policy is, the more ethically justifiable it is, other things being equal; and conversely, the more demanding for individuals a coercive public policy is, the less ethically justifiable it is, other things being equal.

Since, arguably, health authorities should always have the strongest ethical justification possible for implementing coercive policies, they should always strive to implement policies that are the least demanding possible for individuals, other things being equal. Thus, public policies should aim at compelling individuals to do what is as close as possible to the ‘easy rescue’ end of the demandingness spectrum, other things being equal. If we think that the demandingness objection as formulated at the beginning is valid, a corollary of the *demandingness-policy* principle is the following:

*The ethics-policy principle*: public policies should aim at coercing individuals into doing only what they have a moral obligation to do anyway, other things being equal; that is, they should coerce individuals into doing what is not so demanding as to fall outside the domain of individual moral obligations. When this is not possible, the demandingness-policy principle applies.

### Principle of least restrictive alternative

These two principles can be compared with the widely accepted principle in public health ethics, namely the principle of ‘least infringement’ or ‘least restrictive alternative’ (see e.g. Saghai 2013; Gostin 2008): public health policies should be the least restrictive possible that are compatible with the realisation of certain valuable goals. Restrictiveness can be understood in terms of the degree of *coercion* exerted by a certain policy. A policy is coercive if, because of external factors, an individual is left with ‘no reasonable choice’ or ‘no acceptable alternative’ (Wertheimer 1989) but to comply with a certain requirement, and thus coerciveness can be understood in terms of influence of a certain proposal (or policy) on a person’s will (e. g. Frankfurt 1973; Feinberg 1989). The higher the penalty for non-compliance (e. g. fines, or withdrawals of benefits, or exclusion from certain services), the more coercive is the policy, regardless of how demanding the requirement is. Obviously, the degree of coercion of a policy is logically independent from the *demandingness* of what individuals are required to do or coerced into doing. I can be heavily coerced into doing something which is very minimally demanding, such as taking fluoride pills to prevent tooth decay or being vaccinated, when there are very heavy penalties associated with failure to do either of this things. I can be very mildly coerced, or not coerced at all (for example, I could simply be nudged), into doing something that is very demanding, such as forgoing antibiotics to treat mild infections. But the logical independence of level of coerciveness and level of demandingness does not imply that they are independent also from an ethical point of view. Quite the opposite, according to the demandingness-policy principle, the demandingness of a certain requirement weakens the ethical case for coercion. Thus, one implication of the principle of least restrictive alternative is that when implementing a very coercive policy, a state ought also to ensure that the requirement is the least demanding possible (within reasonable boundaries). Ideally, according to the ethics-policy principle, a state ought to do whatever it reasonably can to ensure that the requirement coincides with an independent moral obligation of individuals — although, as we shall see, in practice this is not always possible.

### Consequentialist and non-consequentialist demands

Often, the demandingness objection and the ‘duty of easy rescue’ are associated with consequentialist, and specifically utilitarian, ethical theories (see e. g. Hills 2010 about the demandingness objection; Savulescu 2007 about the duty of easy rescue). Often, it is maximization of utility that is considered overdemanding and the term ‘easy rescue’ refers to the consequences of an action, that is, to the fact that the benefit or harm avoidance that would result from an action (or inaction) demand only a reasonably small individual cost. However, some of the public health policies we have been discussing urge us to rethink the scope of the two concepts beyond consequentialism. While quarantine and isolation can indeed be justified by considerations of harm prevention, since any infected individual could potentially spread the contagion, in the case of vaccination and antibiotic resistance the (moral) demand on individuals is not only and not necessarily that of preventing harm to others — which would be justified on consequentialist grounds and might give rise to a duty of easy ‘rescue’ — but, arguably, also that of making their fair contribution to a collective enterprise. The reason is that often any individual who gets vaccinated does not make a significant difference to whether herd immunity is realised or maintained, and any individual who foregoes antibiotics does not make a significant contribution to whether antibiotic resistance is sufficiently contained. Thus both public health issues are primarily a matter of collective, and not of individual responsibility. Granted, any non-vaccinated individual could potentially infect others, but if vaccination rates are already sufficiently high, this is very unlikely to happen. And any one individual could develop some form of antibiotic resistance that can be passed on to others, making them less responsive to antibiotics (Costelloe et al. 2010), but if such individuals are very few, antibiotic effectiveness would still be significantly contained. Thus, the requirements to get vaccinated or to avoid antibiotics in mild infections do not necessarily have a consequentialist justification, or an exclusively consequentialist justification. The requirement to contribute to public health can in such cases be justified (also) by considerations of fairness. Arguably, individuals have a fairness-based obligation to take on themselves their fair share of burdens entailed by the fulfilment of a collective responsibility, such as the collective responsibility to realise herd immunity (Giubilini et al. 2018; Giubilini 2019). But also requirements that do not have a consequentialist justification can be very demanding and even overdemanding — indeed, the demandingness objection is not a prerogative of consequentialist theories (see e. g. Ashford 2003). Requirements of fairness are an example. Getting vaccinated might be a not very demanding moral requirement of fairness, but foregoing antibiotics in the case of treatable mild infections is quite a demanding requirement of fairness, and perhaps so demanding that it falls outside the domain of moral obligations. Arguably, if you can make your fair contribution towards a public good like herd immunity at a very small cost to yourself, you morally ought to do it. In this paper we talk of ‘easy rescue’ for brevity’s sake, but with that label we will refer to any moral duty that is made very stringent by the fact that it involves a very small individual cost and by the fact that something sufficiently valuable is at stake, be it harm avoidance (as in the standard case), or fairness, or something else.

### Beyond easy rescue

#### comparative vs absolutist accounts of demandingness

Of course, there are moral obligations that remain *moral* even if they extend beyond a mere duty of easy rescue, and that therefore justify coercing people into fulfilling them when there is something sufficiently valuable at stake. The duty of easy rescue can be taken to be an *uncontroversial* moral duty, in the sense that everybody should agree to it, no matter what ethical theory or ethical view they subscribe to (although there would still be disagreement on whether the duty is merely that of preventing harm, or of benefiting others, or of making one’s fair contribution, or something else). But this does not mean that there are no moral duties when the requirement is more demanding than a mere easy rescue. Moral duties can be very demanding in certain cases while remaining moral. For instance, most would agree that I have a moral (and indeed the legal) duty to stop and assist a person who needs help and whose life is at risk if I can reasonably do that, even if doing so costs me something valuable to me, such as missing an important appointment or a flight. Arguably, this moral duty is more demanding than a moral duty to be vaccinated, but it remains a moral duty nonetheless. But precisely how demanding can moral duties be, before they cease to be ‘moral’? While we do not have the ambitious aim of answering such question, we provide here two possible approaches to it.

On some accounts, the demandingness of *moral* obligations might depend not only on the personal costs involved, but also on the value of what is at stake. We can call this a comparative account of demandingness. A requirement that is very demanding in absolute terms can still be reasonably or minimally demanding in relative terms, and therefore it can still be a moral requirement, assuming what is at stake is sufficiently valuable. This understanding of demandingness is expressed in Peter Singer’s principle that ‘if it is in our power to prevent something bad from happening, without thereby sacrificing anything of *comparable* moral importance, we ought, morally, to do it’ (Singer 1972, p. 231). According to this view, very large sacrifices are *morally* required to prevent even greater harms. If we couple this principle with our ethics-policy principle, it follows that if what is of ‘comparable moral importance’ with respect to our sacrifice is also something that a state has the responsibility to protect, then the state is justified in coercing individuals into making that sacrifice even when that sacrifice is very large, because making that sacrifice would still constitute a moral obligation for the individual.

However, many people would endorse what we might call an absolutist account of demandingness and of the demandingness objection, that is, they would put an upper limit to the level of demandingness that can plausibly characterize a moral obligation, beyond which an individual is under no moral obligation to rescue, no matter what is at stake. To borrow from a classical example in moral philosophy, I might even have a moral duty to donate blood if that was the only way to save a person’s life, but I certainly do not have a moral duty to sacrifice my life in order to save, say, five people, for example by donating one of my organs to each of them in case each of them needed a different organ to survive. There might be disagreement about where exactly to draw the line — many would argue that I don’t have an obligation to donate a kidney to a stranger — but many people would agree that there is an absolute line to be drawn somewhere.

Because we do not have space here to argue extensively in favour of either account of demandingness and of the demandingness objection, we will assume here the least controversial one, that is, the absolutist account.

### Demandingness

#### intuitions and subjectivity

Basing the legitimacy of coercive policies on demandingness can be problematic for at least two reasons.

First, whether and to what extent something is demanding or overdemanding and whether the degree of demandingness is consistent with the existence of a moral obligation is in large part a matter of intuition. But every time we rely on intuitions to make certain judgments — any kind of judgement, not necessarily moral ones — there will be cases in which intuitions are not clear. Such vagueness makes it sometimes difficult to decide whether something is a moral duty, at least according to the standard understanding of demandingness and supererogation in terms of cost to the individual (Ackeren 2018): is foregoing antibiotics and therefore enduring a mild but painful infection for longer too demanding to be a moral obligation and to be legitimately coerced? And what kinds of infections, and in what circumstances, precisely make foregoing antibiotics very demanding or too demanding to be a moral obligation?

Second, the degree of demandingness depends on personal values and subjective experience: the same requirement can be differently demanding to different individuals, depending on personal circumstances, including psychological factors and moral or religious beliefs. Staying in bed three additional days because of an infection might pose different costs to different individuals, and costs of different nature (psychological, economic, social, etc.), depending on personal circumstances. Being vaccinated might be cost free and indeed beneficial to most people, but not to those who have very strong moral or religious opposition to vaccines (and the same could be applied to other medical interventions, e. g. blood transfusions) Such variability in the experience of demandingness creates obvious problems when we use demandingness as a criterion for determining moral and legal obligations.

Both problems suggest that we need to have some objective, or at least intersubjective, parameter of what counts as demanding and overdemanding that can reasonably be agreed upon in order to establish moral and legal obligations. In other words, it is certainly important to distinguish between subjective and objective costs of different requirements, but also to acknowledge that for the purpose of determining what counts as an overdemanding legal requirement, it is the objective cost that matters. Of course, one could endorse some form of ethical subjectivism whereby it is up to any individual to determine what does and does not count as a moral obligation for herself, for example depending on how demanding it is for them to act against certain ethical or religious beliefs. However, this would not solve the problem with regard to policy making: a criterion for what counts as too demanding would still be required. While there certainly is a subjectivist understanding of demandingness, what we are interested in here is a notion of demandingness that is meaningful and useful for the purpose of reasonable policy making. Admittedly, determining exactly what this parameter is could be very difficult. Inevitably, there would be cases in which a certain judgment of demandingness will be imposed on individuals which contrasts with their subjective experience or their intuition. But this is a cost that cannot be avoided, and it is in fact not a prerogative of public health policies. Speed fines, taxation, airport security are just a few examples of policies where what it is reasonable to demand of people is simply assumed on the basis of a reasonable standard, that is, a standard on which most people would intuitively agree, even if in certain cases the intuitions are not so firm or widespread and even if they impose a very high subjective cost on some individuals. Besides, if the subjective costs were to determine the level of demandingness of a certain legal requirement, then basically any requirement could become overdemanding for some individuals, thus making the concept of demandingness useless or even meaningless.

However, while the demandingness in terms of subjective costs should not affect the degree of legitimate coerciveness of public health policies, there is a way to make sure that public health policies are not too demanding also in the subjective sense, and therefore to give subjective costs at least some consideration in policy making. As we explain in Section 4, subjective costs may inform opt-out procedures in cases in which exemptions would not compromise the relevant public health good at stake (e. g. herd immunity). What we call ‘hard opt out’ mechanisms would allow individuals to be exempted from coercive measures that they find too demanding in a subjective sense — what we can call ‘conscientious objection’ — through demanding exemption procedures, such that individuals would likely opt for the exemption only if the policy implied a significantly high subjective cost.

## 3 Implications for three public health policies

Let us try to draw a few practical implications from the discussion of the previous section. We will discuss the three cases in public health we have mentioned in the introduction, and to which the considerations of the previous section can be applied. For each of them, we will propose a type of policy solution that is not only ethically justified, but the most likely to be effective. We cannot rule out, however, that less restrictive policies such as nudging would work equally well. But as we suggest towards the end of the paper, whether such alternatives policies are all things considered ethically preferable depends on a number of additional ethical considerations (e. g. fairness) whose discussion is beyond the scope of this paper.

### Vaccination case

If it could convincingly be argued that getting vaccinated or vaccinating one’s children is a moral duty of easy rescue, there would be a strong case for implementing coercive vaccination policies in virtue of the ethics-policy principle. In other words, if being vaccinated is an uncontroversial and basic moral duty of easy rescue, and considering how important for the collective the good of herd immunity is, there is a strong case for state interventions that punish those who don’t fulfil their moral duty. Arguably, not only is vaccination a moral duty of easy rescue (Giubilini et al. 2018), but it also entail significant benefits to the individual. Therefore, the justification for state coercion is very strong. Not only is vaccination not harming individuals, but it benefits them.

What about the subjective experience of demandingness? Vaccination is normally an easy rescue, as we have defined it here, but if someone has very strong ideological (e. g. religious) opposition to vaccines, or is very scared of possible side effects (which are extremely rare), then vaccinating one’s children would probably be more demanding than in the case of someone who, for instance, is only slightly concerned about the side effects of vaccines or is convinced that vaccines are beneficial. Still, this additional psychological burden does not imply that there is no moral duty to vaccinate on grounds of overdemandingness. It seems reasonable to suppose that the reasonable threshold for overdemandingness, that is, the threshold that determines whether something is a moral obligation, is well beyond the level of such psychological burdens, for three reasons. First, it would be very rare that someone’s ideological opposition to vaccines is so strong as to make vaccination overdemanding: people contradict the prescriptions of their religion, even the core ones, very frequently. Second, because of the distinction between subject and objective costs discussed above, we do not consider subjective costs that relevant in many other areas of morality and of public policy, and therefore there is no special reason for doing that in the area of vaccination ethics and of public health policy. To compare, if religious practices involved cruelty to animals, they would legitimately be banned. In the case of vaccination, one objective and plausible standard to determine the degree of demandingness is a simple cost-benefit analysis in medical terms: the medical benefits of vaccination do outweigh the medical costs, even when people perceive otherwise. Third, the perceived individual moral obligation to stick to one’s ideological opposition to vaccines conflicts with other moral obligations that can be established on more solid and commonly agreed upon grounds, such as the moral obligation to protect one’s children from vaccine preventable infectious diseases and the moral obligation of fairness to make one’s contribution to herd immunity. Thus, it can plausibly be argued that vaccination is not overdemanding on any understanding of the term that is meaningful for the purpose of policy making (though it might be on a subjectivist understanding of demandingness), that indeed it is a duty of easy rescue, and that therefore a state has a strong justification for implementing coercive vaccination policies. However, as we explain in Section 4 below, some subjective costs such as very strong moral or religious opposition to vaccines may justify some forms of opt-out mechanisms, provided these do not compromise the relevant good in question (e. g. herd immunity), thus giving some consideration to subjective costs in policy making.

### Antibiotics

Antibiotic resistance (ABR) constitutes a ‘global health security issue’ according to some (Balasegaram et al. 2015). As said above, to contrast ABR, we need, among other things, to reduce the amount of antibiotics *collectively* consumed (O’Neill 2016), so that they can retain their effectiveness when they are most needed. Ideally, reduction in consumption needs to happen at the global level, which requires internationally coordinated effort (Hoffman and Ottersen 2015), also involving countries which present specific problems such as those where people can access antibiotics without prescriptions (e. g. India) and those where, on the contrary, access to antibiotics is still difficult. Some have argued that there is a need to reduce consumption in high-income countries (HICs) and to slow down the growth in consumption rates in low- and middle- income countries (LMICs) (Klein et al. 2018). This is also because some countries, particularly LMICs, have not so far enjoyed their fair share of benefits of antibiotics and are less responsible for ABR, and therefore it would be unfair to require them to reduce antibiotic consumption to the same extent as other countries, particularly HICs (though this consideration is not generalizable as some LMICs like India are among the main contributors to ABR due to widespread and unregulated use of antibiotics). Establishing which countries may be legitimately (that is, fairly) burdened with policies aimed at reducing antibiotic consumption is beyond the scope of this paper. Here, we will only focus on local policies to reduce antibiotic consumption in those countries where those policies can be deemed fair.

One possible scenario is that satisfactory containment of antibiotic resistance would require leaving certain infections untreated in otherwise healthy individuals (Foster and Grundmann 2006), or not prescribing antibiotics where there is a low probability of bacterial infection (throat infections with viral clinical manifestations) so as to ensure that effective antibiotics are available when they are seriously needed. For example, mild and self-limiting infections, e. g. urinary tract infections in otherwise healthy patients. might be a good target for antibiotic stewardship programs. However, this would impose some discomfort and inconvenience, as well as entailing a small risk of escalation of infection. It might be the case that in order to preserve the common pool resource of antibiotic effectiveness, some significant risk might need to be imposed on individuals. In all such cases, what we would be requiring individuals to do is more than minimally demanding, to the point that it is at least doubtful that individuals have a moral obligation to do it. Perhaps it would be unreasonable to say that those who take antibiotics are acting immorally. In such cases, individuals would have strong enough non-moral reasons to use antibiotics anyway (such as the self-interested desire to recover quickly), and no moral reason to forego antibiotics. Therefore, according to the ethics-policy principle, it seems that the state is not justified in simply coercing people into foregoing antibiotics. Instead, individuals need to be given stronger non-moral reasons to forego antibiotics, so as to make it a reasonable option for them. To the extent that the promotion of the health goods in question falls within their responsibilities, governments have an institutional responsibility to use incentives to encourage the desired behaviour. An implication of the different level of demandingness involved is that in the case of foregoing antibiotics, unlike the case of vaccination, states might have to accept the fact that some people would choose to take antibiotics and forego the incentive, as long as the number of these people is sufficiently small, a condition that could be guaranteed by making incentives large enough. Incentives, contrary to fines and other forms of punishments, increase individual freedom.

One important feature of incentive-based policies is that people would remain free to decide whether or not to choose the incentivised option. According to some, however, also very generous offers such as large incentives, and not only threats of penalties, can be coercive (Frankfurt 1973, p. 79; Held 1972, p. 57) if they are unreasonable to refuse. We do not agree with this view, although we do not have the space to argue against it here (but see e. g. Savulescu 2001). Those who think that large offers can be coercive would need to say that incentive-based policies for demanding requirements should ensure that the incentive is not so large as to represent an undue inducement and therefore to exert a too high degree of coercion, but at the same time not so small as to provide only very weak non-moral reasons to fulfil the requirement, which would render the policy ineffective. However, we want to emphasize what we take to be the real risk of incentive-based policies: not coercion, but exploitation (Savulescu 2015). Providing too small incentives increases the risk that people in dire conditions might accept the incentive even when it is not proportionate to the sacrifice they are required to make. In this scenario, they would be taken advantage of in virtue of their vulnerable situation, when they would otherwise not have accepted such a small incentive. So any incentive needs to be provided on the basis of a fair minimum that prevents it from being exploitative — and of course anything above the fair minimum is a bonus. Also, importantly, incentive-based antibiotic policies raise the issue of what level of risks individuals may permissibly be incentivised to accept, and how large incentives should be on the basis of the level of risk (ranging from the risk of staying in bed a few additional days to the risk of death). While we do not have the space to tackle this issue here, it is important to point out that any incentivebased public health policy would need to determine an adequate ‘price’ for the level of individual risk imposed.

Indeed incentives need not be only financial. They could include more vigorous monitoring, access to other relevant health care goods, or social recognition and praise.

### Quarantine/isolation

What about measures such as quarantine and isolation? While quarantine separates people who have been exposed to an infectious disease, isolation separates sick and contagious individuals. Forced quarantine and isolation are sometimes necessary to reduce the risks posed by certain infectious diseases, as was the case in the SARS outbreak in 2003 and the Ebola outbreak in West Africa in 2014-15. Both measures involve infringements upon individual rights of freedom of movement and association. Quarantine and isolation certainly are vastly more demanding than vaccination and probably, in most cases, also more demanding than foregoing antibiotics for certain mild and self limiting infections, especially when they have to take place in poor countries. During the 2014-15 Ebola outbreak in West Africa, for instance, quarantine and isolation measures entailed stigmatization and loss of livelihoods (Calain and Poncin 2015), but also disruptions of communication with family members and of access to sanitation facilities and clean water (ACAPS 2016; Kutalek et al. 2015). Also, and importantly, those in quarantine are at greater risk of contracting infections from other exposed individuals who have been infected.

The relevant aspect of quarantine and isolation is that they potentially prevent any single individual from causing harm: any single individual infected could have a devastating impact on other individuals and on public health by spreading the infection. Therefore, it seems that where quarantine and isolation measures are required, they have to be imposed on each individual because each individual could ‘make a clear difference’. Even if the requirement is very demanding, no exception can be made, and therefore individual liberty needs to be restricted. Thus, even if the high level of demandingness would require the use of incentives, a mere system of incentives — like the one we have argued should exist in the antibiotics case — is not sufficient because — unlike the antibiotic case — people cannot be left free to decide whether or not to choose the incentivised option.

At the same time, a merely coercive system based exclusively on penalties — like the one we have argued should exist in the vaccination case — is not ethically acceptable, because quarantine and isolation are not independent moral obligations, at least if we accept the demandingness objection and the absolutist account of demandingness. The most ethical solution in this case is a compromise between the two preceding solutions: it is necessary to use a high degree of coercion to ensure that quarantine and isolation policies are effective, but at the same time the state ought to properly compensate individuals for the fact that they are fulfilling an overdemanding legal obligation.

Ideally, the size of the compensation should be responsive to the difference between what individuals are actually required to do by law and what they would have an independent moral obligation to do (e. g. avoid unnecessary physical contact with other people): the larger such difference, the larger the compensation, given that it is precisely that difference that grounds the ethical obligation of the state to compensate. Compensation might be financial in nature, but might also involve provision of other kinds of benefits or services, such as priority in accessing needed medical treatment in the future, subsidisation for medical services, or the building of infrastructures in villages where a high percentage of the population is confined.

Importantly, a necessary, though not sufficient, part of the compensation for fulfilling an overdemanding requirement includes the realisation of those basic conditions that would ensure that quarantine and isolation approximate as close as reasonably possible an ‘easy rescue’: food, clean water, sanitation, means to communicate with loved ones are all conditions that a state ought to guarantee when implementing coercive quarantine or isolation measures, in order to ensure that the policy is the most ethically justified possible (Giubilini et al. 2017). Any compensatory measure should assume this as a baseline and build upon such basic goods in proportion to the demandingness of the imposition.

The concept of reciprocity in public health (Viens et al. 2009; Silva and Smith 2015) could be used to justify compensatory measures in the context of public health policies: when a state requires people to do something supererogatory, that is, too demanding, such as submitting to quarantine or isolation measures, the state owes something in return to these people as a matter of reciprocity. Our discussion is consistent with this principle of reciprocity in public health: according to our argument, it is because individuals are not independently morally required to fulfil a certain requirement that they are owed something in return.

However, it is important to clarify two aspects of reciprocity in light of our ethics-policy principle. First, we need to be clear as to when the principle of reciprocity should be applied. According to Diego Silva and Maxwell Smith,

reciprocity maintains that when an individual is subject to a limitation on their human rights or freedoms for the sake of a public health emergency, the State must support and compensate that individual for his or her loss, so they are not unduly harmed.

(Silva and Smith 2015, p. 53)

This is not quite precise, because not *any* limitation of freedoms as such justifies compensation — it all depends on whether individuals would have an independent moral obligation to give up their freedom for the sake of the collective good. For instance, compulsory vaccination does constitute a limitation of freedom but does not call for compensation, according to our argument.

Second, we need to be clear as to what exactly reciprocity demands. Again according to Silva and Smith, the principle of reciprocity demands that ‘society provides resources such as food and water to those burdened by restrictive measures like isolation or quarantine’ (2015, p. 54); while this is correct, in many cases such measures would not be sufficient to fulfil a state’s ethical obligations. While these might make the sacrifice less demanding, the requirement might still be very demanding as there are problems beyond food and water supply that would make isolation and quarantine very burdensome, such as social isolation and limitation of freedom of movement. In such cases, other forms of compensation (including perhaps financial ones) would be ethically required. Reciprocity requires that the form of compensation adopted be proportionate to the sacrifice requested. As Viens and colleagues rightly note, ‘[r]eciprocity requires that one return the good one has received, or responds to harms performed, in a fitting manner’ (Viens et al. 2009, p. 211). Our argument suggests that what constitutes a ‘fitting’ manner is some proportionality between the demandingness of a requirement and what is owed to those who submit to that requirement.

It is also worth noting that incentives and compensation are not mutually exclusive. For example, in the antibiotic case, it seems that people who are incentivised to forego antibiotics for certain infections are also entitled to compensation if they are harmed. This is because the existence of incentives does not necessarily detract, or does not detract significantly, from the demandingness of a certain requirement. For example, a prolonged infection could have an opportunity cost (economic, social, or other kinds of costs) that needs to be compensated for as much as possible, and that might be only partially mitigated by the incentive.

## 4 Regulating public health policies

### A guiding table

In the table below we provide a schematic overview of how our main philosophical claims apply to different possible public health policies. We have italicized those we have discussed in the paper, and made other suggestions, the position of which in the table has not been discussed in this paper and might require further ethical analysis.

One might argue that when a certain requirement is not demanding, but does not entail any individual benefit either, there are ethical reasons to prefer incentives over coercion, in order to comply with the principle of least restrictive alternative (and perhaps resort to coercion only if incentives are ineffective), given that incentives are not coercive. This claim can be grounded in a variation of the demanding-ness-policy principle, according to which a policy should be the less restrictive possible, not only the more demanding a policy is for an individual, but also the less beneficial it is for the individual on which the requirement falls. While we have not discussed here whether the existence of individual benefits should make a difference to the legitimate level of restrictiveness of public health policy, we take this to a very plausible principle. After all, if the demandingness of a certain policy is determined by the costs on the individual, and if we assume that what matters for determining level of demandingness is the net cost given by the total cost minus any individual benefit, it seems that the demandingness-policy principle does imply that benefits strengthen the case for coercion, thus justifying more coercive measures than would be the case if there were no individual benefit. It is important to point out that we are only concerned here with policies justified by significant public health interests. Individual benefits of such policies for those who are subject to them might play a role in determining the legitimate degree of coercion when coercion has a justification in terms of public health, but do not by themselves provide sufficient justification for coercive policies, that is, on paternalistic terms.

At the same time, however, a certain requirement can remain minimally demanding even in the absence of benefits, thus justifying at least some degree of coercion in the absence of benefits, rather than incentives. For this reason, in the table below we have distinguished between two forms of coercion, what we call ‘soft’ and ‘hard’ coercion, whose respective justification depends on the extent to which the requirements they pose benefit individuals. In this context, what makes coercion ‘soft’ or ‘hard’ is simply the size of the penalty for noncompliance. We have proposed that, for example, deceased organ donation — an example of requirement that is not demanding to an individual, but does not benefit the individual either — should preferably be promoted through coercive policies that are softer than, say, vaccination policies, given that vaccination is beneficial to the individual but deceased organ donation is not. Thus, giving non-organ donors lower priority on waiting lists in case they themselves need a transplant would be a form of coercion that would put some pressure on individuals to sign up for deceased organ donation, thus exerting some coercion, but not as much coercion as vaccination policies with very high penalties for non-compliance, such as preventing non-vaccinated children from enrolling in schools or having very high legal penalties for non-vaccination; the chances that a healthy person would need an organ transplant are quite low, and with a policy that is effective at significantly increasing organ supply the chance that this person would not get the organ they need because of lower priority are very low. Quitting smoking can be in itself quite demanding but also very beneficial, so that the objective cost is overall relatively small and justifies hard forms of coercion (e. g. heavy taxation). Granted, the distinction would need to be more fine-grained than that: within both categories there would be different degrees of legitimate coerciveness, on the basis of how demanding and how beneficial to individuals each policy is (for instance, quitting smoking is normally more demanding than not starting to smoke), and the very same boundaries between hard and soft coercion would be difficult to trace.

Another way to make the table less controversial and more acceptable to the public would be to give some consideration to subjective costs in the case of coercive policies that are not demanding in the objective sense, and for which hard coercion would otherwise be justified on the basis of the demandingness-policy principle. We can think of a form of ‘hard opt-out’ or ‘hard-nudging’ such as burdensome exemption procedures. Many forms of coercion can already be conceived of as kinds of ‘hard nudging’, in the sense that often people have the option of paying the fine or any other penalty if their opposition to a certain requirement is strong enough, and therefore the (subjective) cost on them of fulfilling the requirement is larger than the cost posed by the penalty or the disincentive. But other forms of hard nudging could be considered. For instance, some (e.g. Navin and Largent 2017) have suggested that nonmedical exemptions to vaccine mandates should be granted only through relatively complex bureaucratic procedures and quite demanding requirements (e. g. attending information sessions about vaccines’ benefits), in order to ensure that exemptions are granted on the basis of sincerely held moral or religious beliefs that would be very costly for individuals to give up, rather than for reasons of mere convenience. This would be a good way of ensuring that the subjective costs individuals would pay in fulfilling a certain requirement is not too large. If people are prepared to do alternative demanding things to avoid fulfilling a certain requirement, it means that fulfilling that requirement would have been very costly for them, even if only in a subjective sense. This kind of ‘conscientious objection’ policies can minimize demandingness and at the same time respect individual autonomy, but it is important to point out that they are only warranted if they do not compromise the public health good that make coercive public health policies legitimate in the first place (e. g. the exemptions are not too many).

It is also important to bear in mind — though this should be obvious — that the principle of least restrictive alternative is just one principle among others, and not an absolute one. Thus, sometimes a more coercive measure can be ethically preferable to a less coercive one, in virtue for example of a principle of fairness in allocation of scarce resources combined with the ethics-policy principle or the demandingness-policy principle. For instance, when a less coercive policy would be more costly to implement than a more coercive one, when this additional cost would use resources that could be used for valuable purposes, and when individuals have an independent moral obligation to do a certain thing, then the more coercive policy might be ethically preferable.

Also, importantly, by no means do we intend to suggest that the three policy options here discussed — coercion without compensation, incentives, and coercion with compensation — exhaust the range of all possible public health interventions. There is a range of policy options, well exemplified by the Nuffield Council on Bioethics’ ‘intervention ladder’ for public health interventions, that goes from simply providing information and persuasion at the non-restrictive end, to outright compulsion at the most restrictive one (NCB 2007). To mention just one possible alternative type of policy, nudging is a type of non-coercive and non-restrictive public health intervention that has received lot of attention in public health ethics recently; where nudging is effective, a principle of least restrictive alternative suggests that it is preferable to coercion, other things being equal, where other things include any ethically relevant consideration in addition to effectiveness and liberty, e. g. fairness. How to balance the values of freedom, effectiveness, and fairness is a very important issue in public health ethics, but it goes beyond the scope of a paper focussed on the ethical relevance of demandingness. In any case, it is perhaps worth noting that where individuals have strong beliefs or feelings (e. g. ideological opposition to vaccines or to deceased organ donation) or strong interests (e. g. in their freedom of movement) against certain things, it is unlikely that nudges will be effective.

Thus, in the table below we present the policy types that we have considered in this paper as well as a few others, but in virtue of the considerations just made, we are aware that the picture is way more complex than this. It would be difficult to justify coercive policies without compensation when the requirements on individuals are very demanding, which explains why two of the three boxes in the ‘coercion without compensation’ column are empty, and there seems to be no justification for incentivising individuals to fulfil requirements that are minimally demanding, which is why the first box in the ‘incentives’ column is empty.

## 5 Conclusions

Ethics can be very demanding, but it cannot be too demanding, at least on most understandings of ethics and according to the demandingness objection. Public health policies can be very demanding as well. Foregoing antibiotics and quarantine or isolation seem to be requirements that are too demanding to represent *independent* moral obligations. However, such measures can be necessary for public health reasons. The most ethical solution, we have suggested, is to acknowledge and mitigate the demandingness of certain public health policies by implementing a system of incentives and/or compensations: the former provide individuals with non-moral reasons to do something supererogatory (with the size of the incentives proportionate to the level of harm or risk of harm imposed), and the latter compensate individuals for being forced to do something supererogatory.

Conversely, when the requirement falls within the domain of independent moral obligations of easy rescue, and the good at stake is sufficiently important and is one that the state has the responsibility to ensure, the state is justified in using coercive measures. One such example is vaccination. In such cases, not only is the state under no obligation to provide incentives or compensation, but it is morally justified in punishing those who do not fulfil undemanding requirements such as getting vaccinated, given that these people are simply failing to fulfil some independent moral obligations. This occurs in Australia where vaccine refusers are denied family financial benefits (‘no jab, no pay’) or childcare (‘no jab, no play’).

Demandingness has important implications for ethics, but — so we have argued — also for policy making, and particularly for public health policy. Sometimes, for the sake of public health, it is necessary to require people to do very demanding things, and sometimes it is necessary to require people to do things that are only minimally demanding. In either case, good policy making should take the level of demandingness into account, and use carrots and sticks accordingly.

## Figures and Tables

**Table 1:** Demandingness of Policy Types.

	HARD COERCION (without compensation) with ‘hard opt out’	SOFT COERCION (without compensation) with hard opt-out	INCENTIVES	COERCION with COMPENSATION
**LOW DEMANDINGNESS**	*Vaccination;* wearing seat belts; smoking in indoor public spaces; not starting to smoke (usually disincentivised through taxes); quitting smoking	deceased organ donation; blood donation		
**HIGH DEMANDINGNESS**			*Restrictions on antibiotic prescriptions (with different types of incentives based on the level of individual risk imposed);*	Restrictions on antibiotic prescription (if incentives are ineffective, with the level of compensation based on the risk imposed);
**VERY HIGH DEMANDINGNESS**			Living organ donations	*Quarantine and isolation*

## Footnotes

1 In this paper we will assume this understanding of the demandingness objection and that individuals have reasons to behave morally, but not to go beyond what morality requires. On a different understanding of the demandingness objection (see e. g. Cullity 2003), morality can be too demanding, and when this is the case we do not have reasons to act morally. Deciding which understanding is the most plausible would require a philosophical analysis on the nature and limits of morality, which is beyond the scope of this article. For the purposes of the present discussion — that is, analyzing the implications of demandingness for policy making — this distinction is however not relevant, because what matters is simply the principle that legally enforced requirements should be the least demanding possible, whether or not the requirement is taken to fall within the domain of ‘moral’ requirements. But it seems to us that morality would be quite an anaemic concept if it prescribed us things that we do not have sufficient reasons to do, although on some accounts ‘it is a mistake to think that because [a] theory has implications that only saints will be able to met, it should not be taken seriously’ (de Lazari-Radek and Singer 2014, pp. 323f.). In any case, those who endorse the idea that morality can be too demanding, and therefore that there can be sufficient reasons not to act morally, can simply replace expressions such as ‘moral obligations’ and equivalent ones with phrases such as ‘obligations that are not too demanding’; and those who, following de Lazari-Radek and Singer (2014), think that also very demanding moral theories should be taken seriously, will simply set the bar for what is to be considered too demanding higher than we do in this paper.
